# Synthesis of a Novel Benzoyl Adenosine Analog Containing a 1, 4-Dioxane Sugar Analog and the Synthesis of a Corresponding Uracil Adenine Dinucleotide

**DOI:** 10.3390/molecules16053985

**Published:** 2011-05-12

**Authors:** Qiang Yu, Per Carlsen

**Affiliations:** Department of Chemistry, Norwegian University of Science and Technology, 7491 Trondheim, Norway

**Keywords:** nucleoside analogs, heterocyclic, adenosine, 1,4-dioxane, uracil-adenine

## Abstract

Adenosine analogs in which the sugar unit was replaced by a 1,4-dioxane sugar equivalent, were prepared by coupling the 1,4-dioxane sugar analog as its anomeric acetates, with *N*6-benzoyl protected adenine. The 1,4-dioxane system was obtained in an enantioselective synthesis from (*R,R*)-dimethyl tartrate. Using standard phosphorimidite methodology, the adenine analog was further reacted with a 1,4-dioxane uridine analog to give the corresponding, protected dinucleotide, set-up for further condensation into larger oligonucleotides.

## 1. Introduction

Pursuing development of new antiviral and antitumor agents, a number of new nucleosides analogs have been synthesized in which of the sugar structures were modified [1]. The sugar unit has for example been replaced by a 1,4-dioxane moiety [2,3,4,5,6]. Some of these structures were reported to exhibit interesting biological activities [2,3], which may be ascribed to their particular flexible conformational properties [4]. Earlier we reported the synthesis of new optically active uridine analogs **1a** or **1b** where the sugar was substituted by an optically active 1,4-dioxane moiety [7]. These uridine analog was further elaborated into the corresponding dinucleotide using standard phosphorimidite methodology [8]. However, we also reported [7] that preparation of the related adenosine analog failed. In this communication we wish to report our recent findings dealing with the synthesis of adenosine analogs **2a **and **2b**, which as the *N*-benzoyl protected compounds were then tested in dinucleotide formation with uridines **1a**.

**Figure 1:**
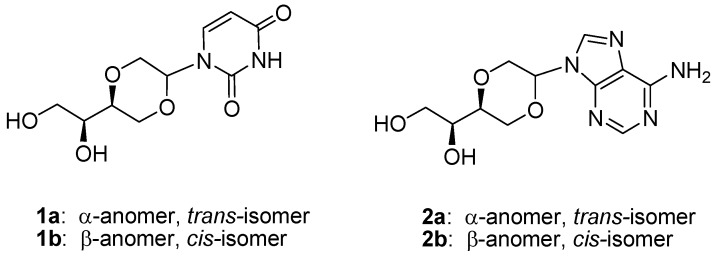
Uridine and adenosine analogs containing a 1,4-dioxane sugar equivalent.

## 2. Results and Discussion

**Scheme 1:**
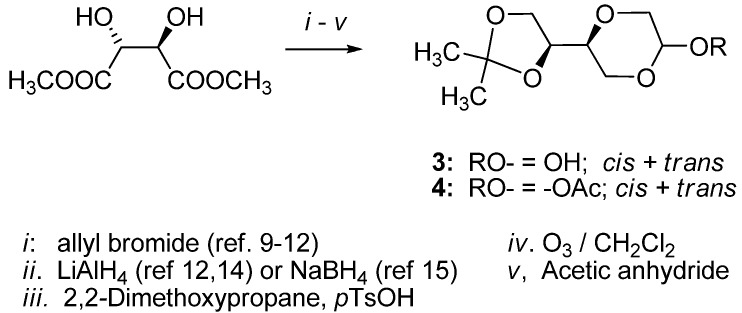
Synthesis of 1,4-dioxane sugar analogs 3 and 4 [7].

The synthesis of 1,4-dioxane sugar analog **3** from (2*R*,3*R*)-dimethyl tartrate was reported earlier [7]. The tartrate was first converted into the corresponding enantiomerically pure diethyl (*2R*,*3R*)-2-*O*-allyltartrate either by the reaction with allyl bromide in the presence of silver oxide or in a tin assisted reaction with dibutyltin oxide [9,10,11]. The allyl ether was reduced by LiAlH_4_ [12,13,14] or NaBH_4_ [15,16] to give a triol which was protected as an acetal with 2,2-dimethoxy-propane in the presence of *p-*toluene sulfonic acid. Subsequent ozonolysis afforded 1,4-dioxane sugar analog **3** which was further transformed into acetate **4 **with acetic anhydride (Scheme 1). All the tartrate stereoisomers are readily available from the chiral pool, conveniently allowing for the synthesis of all the possible stereoisomers of the nucleoside analogs.

### 2.1. Synthesis of Adenosine Analogs

**Scheme 2:**
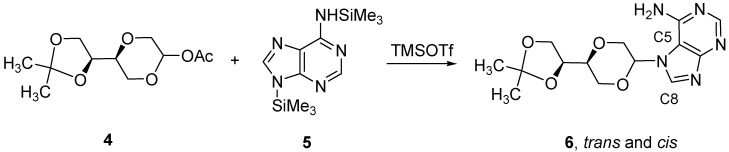
Synthesis of N-7 adenine nucleoside analogs **6**.

The corresponding adenine nucleoside analog from **4** was attempted prepared using a Vorbrüggen procedure [17,18]. Thus, acetate **4** was coupled with silylated adenine **5**, in the presence of trimethylsilyl trifluoromethanesulfonate (TMSOTf) to give **6** as a *cis*/*trans* mixture in 23% yield after flash chromatography. However, HMBC-NMR spectroscopic analysis showed that the product was the undesired *N*-7 regioisomer **6**, as three bond correlations were observed for the anomeric proton with C5 as well as C8 (Scheme 2). 

**Scheme 3:**
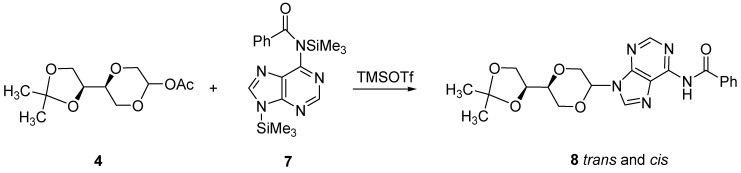
Synthesis of benzoyl adenine nucleoside analogues **8**.

For reasons of forcing the adenine to react at the 9-position, **4** was instead reacted with the silylated *N*6-benzoyl protected adenine, **7**, in the presence of TMSOTf, affording the desired benzoyl protected adenine nucleoside analogues **8 **as a *cis*/*trans* mixture in 42 % isolated yield after chromatographic purification, Scheme 3. The structure was confirmed by NMR analysis. 

**Scheme 4:**
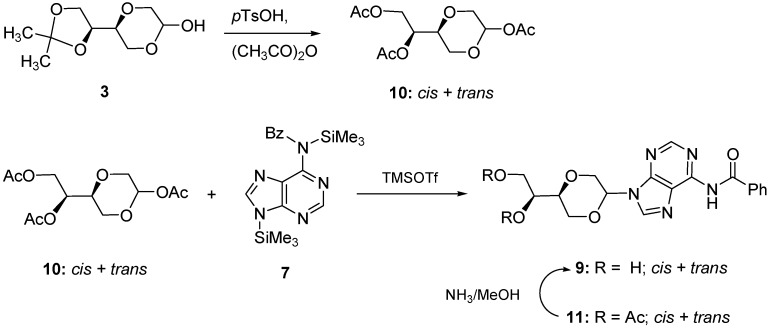
Synthesis of benzoyl protected adenine nucleoside analogue **9**.

Attempts to remove the acetal protection group in **8** failed, as depurination was observed to take place in all cases. A number of literature methods described were tested [19,20], such as cleavage using 80% acetic acid [21] or CSA as catalyst, reaction with Amberlyst 15, HCl [22] or trifluoroacetic acid [23]. Other reagents, such as ferric chloride on silica gel [24], iodine in methanol [25] or alumina or silica gel catalysis in all cases gave no or similar results. The desired benzoyl protected nucleoside analog **9** was finally obtained by the reaction sequence shown in Scheme 4. 

Conversion of **3** to the corresponding triacetate **10** by the reaction with acetic anhydride in the presence of catalytic amount of H_2_SO_4_ [26,27] gave the desired product though in a poor yield. Product **10** was, however, obtained in a satisfactory yield when **3** were reacted with acetic anhydride in the presence of 1.1 equivalent of *p*-TsOH [28]. Subsequently the triacetate was smoothly coupled with the silylated benzoyl adenine **7** in the presence of TMSOTf to afford benzoyl adenine nucleoside analogue **11**. The acetate groups were selectively removed by ammonia in methanol [29] leaving the desired, benzoyl-protected nucleoside analog **9** (Scheme 4). 

### 2.2. Synthesis of Dinucleotide Analogs

**Scheme 5:**
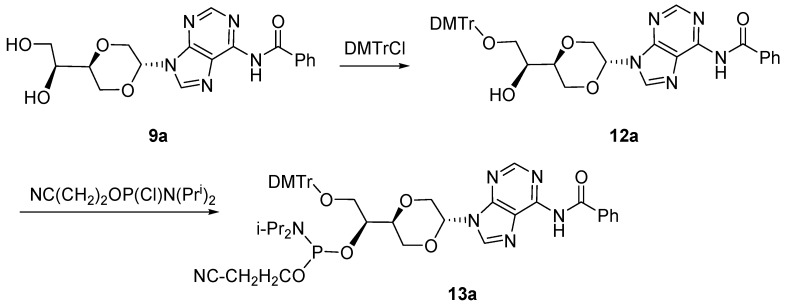
Synthesis of phosphoramidite **13a**.

The pure *trans*-nucleoside analog **9a** was separated by flash chromatography from the mixture of diastereomers of compound **9**. Treatment of **9a** with 4,4'-dimethoxyltrityl chloride (DMTrCl) in pyridine [30] afforded the primary hydroxyl group protected compound **12a **in 40% yield after flash chromatography. Compound **12a** was next reacted with *N*,*N*-diisopropyl-2-cyanoethylphosphor-amidic chloride [31,32] in the presence of *N,N*-diisopropylethylamine to give the desired phosphoramidite **13a **in 51% isolated yield, Scheme 5.

We have previously reported the synthesis of the uridine analog **1**, obtained from acetates **4**. The *trans*-anomer **1a** was isolated in 42% yield after recrystallization from acetonitrile, and subsequently converted to the secondary acetate **14a** [7].

**Figure 2:**
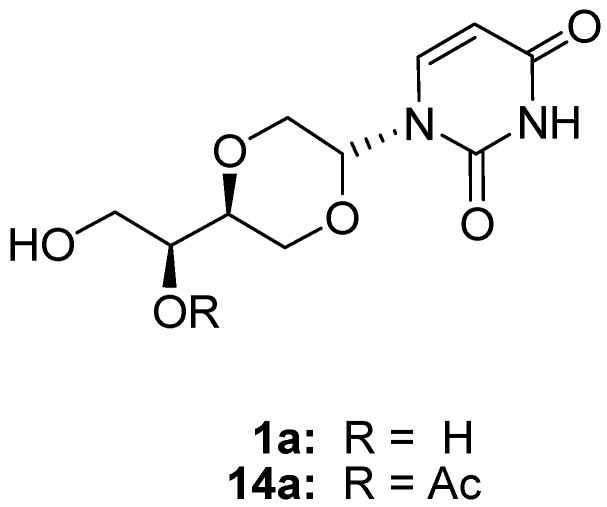
*Trans*-uridine analogs **1a** and **14a**.

**Scheme 6:**
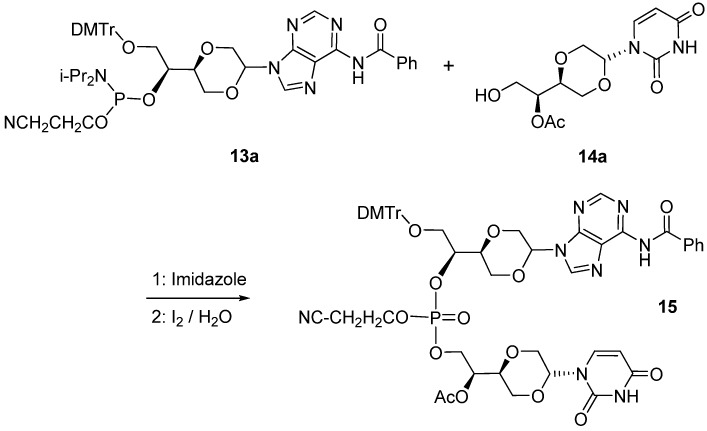
Synthesis of 1,4-dioxane uracil adenine dinucleotide analog **15**.

The coupling of adenosine phosphoramidite **13a** with uridine analog **14a** was carried out in the presence of 1*H*-tetrazole in dry acetonitrile [33], followed by iodine oxidation and purification by flash chromatography on silica gel. This afforded the uracil adenine dinucleotide analog **15 **in 62% yield, Scheme 6. To obtain the analytically pure dinucleotide **15**, the product was purified by multi elution preparative TLC.

Further elaboration of product **15** was not pursued, as it at this stage was demonstrated that the 1,4-dioxane nucleoside analogs can function in the standard reaction scheme for oligonucleotide preparations, used in for example automatic nucleotide synthesis machines. Thus **15** represent a standard intermediate for the further preparation of oligonucleotides.

## 3. Experimental

### 3.1. General

NMR spectra were recorded on Bruker Avance DPX 300 or DPX 400 instruments. Chemical shifts are reported in ppm using TMS (d = 0.0) as the internal standard in CDCl_3_ or relative to 2.50 ppm for ^1^H and 39.99 ppm for ^13^C in [*d*_6_-DMSO] or 3.31 ppm for ^1^H and 49.15 ppm for ^13^C in CD_3_OD. Structural assignments were based on ^1^H, ^13^C, DEPT135 and 2D-spetra, COSY, HSQC, HMBC, and NOESY. EI-Mass and ESI spectra were recorded on a Finnigan MAT 95XL spectrometer. IR spectra were obtained on a Thermo Nicolet FT-IR Nexus spectrometer using a Smart Endurance reflection cell. For ozonolysis was used an OZ-500 Ozone Generator produced by Fishcer Technology. Silica gel Kieselgel 60G (Merck) was used for Flash Chromatography. The solvents were purified by standard methods. All reactions were carried out in inert atmospheres (nitrogen). The synthesis of compounds **1a** and **14a** has been described elsewhere [7].

*(2S,5S)-5-[(4S)-2,2-Dimethyl-1,3-dioxolan-4-yl]-2-acetyloxy-1,4-dioxane* (**4a**) * and (2R,5S)-5-[(4S)-2,2-dimethyl-1,3-dioxolan-4-yl]-2-acetyloxy-1,4-dioxane* (**4b**). To a solution of **3** (1.0 g, 4.9 mmol) in dry pyridine (15 mL) was added acetic anhydride (0.62 g, 6 mmol) at 0–5 °C and the reaction mixture was stirred for 6 hours. The solution was concentrated under reduced *in vacuo* overnight, yielding the crude product in 87% yield as an oily solid material, which was used in the subsequent reaction step without further purification. The anomeric ratio **4a**:**4b** (*trans*-*cis* ratio) was determined to be 4:1 by NMR. The products exhibited the following spectroscopic properties: The *trans*-product **4a** (Figure 1) was assigned the following signals: ^1^H-NMR (CDCl_3_, 400 MHz): δ 1.36, 1.43 (s, 2 × 3H, (CH_3_)_2_C), 2.11 (s, 3H, CH_3_COO), 3.48 (dd, *J* = 8.0 Hz, 11.4 Hz, 1H, H_B1_), 3.63 (m, 1H, H_C_), 3.69 (dd, *J =* 9.4 Hz, 11.4 Hz, 1H, H_D1_), 3.81 (dd, *J =* 6.8 Hz, 8.0 Hz, 1H, (CH_3_)_2_C-O-CH_2_), 3.89 (dd, *J =* 2.6 Hz, 11.4 Hz, 1H, H_D2_), 3.93 (dd, *J =* 2.8 Hz, 11.4 Hz, 1H, H_B2_), 4.00 (dd, *J* = 6.8 Hz, 8.4 Hz, 1H, (CH_3_)_2_C-O-CH_2_), 4.15 (m, 1H, (CH_3_)_2_C-O-CH-), 5.74 (dd, *J* = 2.8 Hz, 8.4 Hz, 1H, H_A_) ppm. ^13^C-NMR (CDCl_3_, 100 MHz): δ 20.9, 25.2, 26.3, 65.2, 65.6, 66.9, 74.2, 74.2, 89.4, 109.7, 169.0 ppm. The protons NMR spectrum of the corresponding *cis*- compound, **4b**, could not be fully assigned due to the peaks overlap with **4a**, however, the carbon NMR spectrum of the *cis*- compound was assigned the following signals: ^13^C-NMR (CDCl_3_, 100 MHz): δ 21.1, 25.2, 26.2, 61.0, 64.9, 67.6, 74.9, 75.1, 88.4, 109.7, 169.8 ppm. The mixture exhibited the following mass spectrum: MS (EI) *m/z*: 247 (M^+^+1), 231(M^+^-CH_3_), 187(M^+^-OAc), 145 (C_6_H_9_O_4_). Elem. Anal. calcd. for C_11_H_18_O_6_: C 53.65, H 7.37; found C, 53.84, H 7.45.

**Figure 1:**
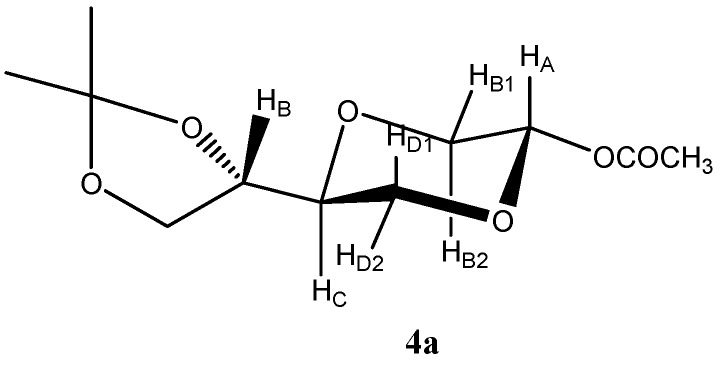
Structure of compound **4a**.

*N-7-{(2R,5S)-5-[(4S)-(2,2-Dimethyl-1,3-dioxolan-4-yl)]-1,4-dioxan-2-yl}adenine* (**6a**) *and N-7-{(2S,5S)-5-[(4S)-(2,2-dimethyl-1,3-dioxolan-4-yl)]-1,4-dioxan-2-yl}adenine* (**6b**). The mixture of adenine (1.35 g, 10 mmol) and ammonium sulfate (124 mg, 0.9 mmol) in hexamethyldisilazane (HMDS, 35 mL) was refluxed overnight. The solvent was evaporated and the residue was dissolved in dry dichloroethane (20 mL). To this solution, compound **4** (0.85 g, 3.5 mmol) was added. The solution was cooled to 0 °C and TMSOTf (0.75 mL, 4 mmol) was added. The solution was stirred for 8 hours at room temperature. The chloroform (50 mL) was added and the solution was washed twice with saturated NaHCO_3_ solution (15 mL). The aqueous phase was extracted twice with chloroform (50 mL), and the combined organic layers was dried over anhydrous MgSO_4_ and filtered and concentrated under reduced pressure. The residue was purified by flash chromatography using a mixture of dichloromethane and methanol (19:1) as the eluent. A white solid (0.26 g, 23%) was obtained which was identified as a 3:2 mixture of the *trans*- and *cis*- products **6a** and **6b**. From the mixture of isomers was extracted the following spectroscopic properties for isomer **6a **(Figure 2): ^1^H-NMR (CDCl_3_, 400 MHz): δ 1.47, 1.48 (s, 2 × 3H, H_I_), 3.87–3.91 (m, 3H, H_B_), 3.97–4.03 (m, 3H, H_A _and H_E_), 4.04 (dd, *J* = 12 Hz, 6 Hz, 1H, H_D2_), 4.07 (dd, *J* = 8.4 Hz, 6.8 Hz, 1H, H_A_), 4.21–4.27 (m, 2H, H_C_ and H_D_), 5.72 (dd, *J* = 8.8 Hz, 4 Hz, H_F_), 5.99 (br.s, 2H, NH_2_), 8.11 (s, 1H, H_G_), 8.54 (s, 1H, H_H_) ppm. ^13^C-NMR (CDCl_3_, 100 MHz): δ 25.2, 26.4, 66.1, 68.5, 60.4, 74.1, 82.0, 110.3, 111.1, 144.0, 151.3, 154.0, 161.6 ppm. For the mixture of **6a** and **6b**: IR (neat): 3418, 3290, 3149, 2979, 2905, 1625, 1595, 1062 cm^-1^. HRMS (ESI): Calcd. for C_14_H_19_N_5_O_4 _[M+Na]^+^ 322.1516, Found 322.1513.

**Figure 2:**
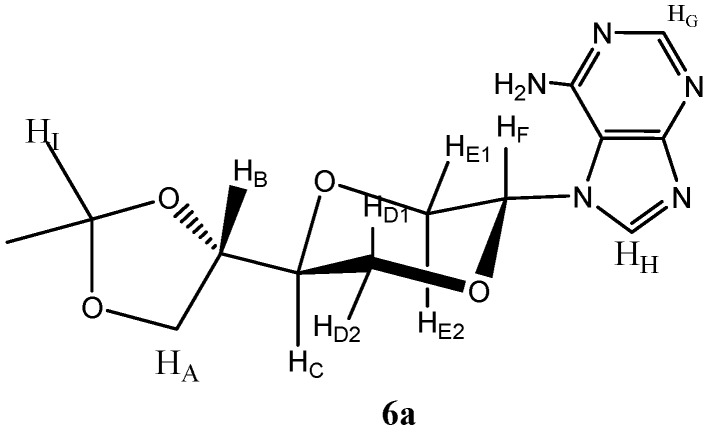
Structure of compound **6a**.

**Figure 3:**
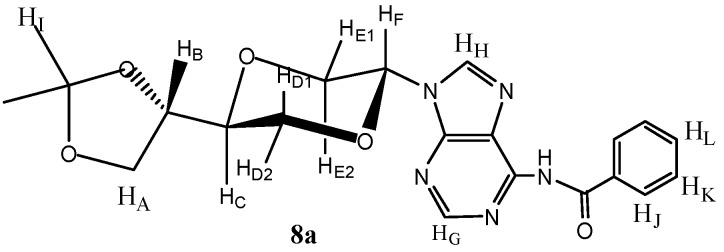
Structure of compound **8a**.

*N-6-Benzoyl-N-9-{(2R,5S)-5-[(4S)-(2,2-dimethyl-1,3-dioxolan-4-yl)]-1,4-dioxan-2-yl} adenine* (**8a**) *and N-6-benzoyl-N-9-{(2S,5S)-5-[(4S)-(2,2-dimethyl-1,3-dioxolan-4-yl)]-1,4-dioxan-2-yl}-adenine* (**8b**). N6-benzoyladenine (2.065 g, 8.7 mmol) and ammonium sulfate (120 mg, 0.9 mmol) in HMDS (50 mL) was refluxed overnight. The solution was cooled and concentrated under reduced pressure. The residue was dissolved in dry acetonitrile (20 mL) and added a solution of **6** (0.77 g, 3.1 mmol) in dry acetonitrile (5 mL). The mixture was then cooled to 0 °C and added TMSOTf (0.6 mL, 3.3 mmol). The mixture was stirred for four hours. Then chloroform (50 mL) was added and the solution washed twice with saturated NaHCO_3_ solution (20 mL). The organic phase was dried over anhydrous MgSO_4_ filtered and concentrated under reduced pressure. The crude product was purified by flash chromatography using a gradient eluent system, first diethyl ether, followed by a mixture of dichloromethane and methanol (19:1) to afford the product as a yellow solid (0.55 g, 42%) as a 3:1 mixture of *trans*- and *cis* products **8a** and **8b**. Pure **8a** was obtained by repeated chromatography. Product **8a** (Figure 3) exhibited the following spectroscopic properties: ^1^H-NMR (CDCl_3_, 400 MHz): δ 1.41, 1.52 (s, 2 × 3H, H_G_), 3.48 (dd, *J* = 11.4 Hz, 9.4 Hz, 1H, H_E2_), 3.79–3.84 (m, 1H, H_C_), 3.94–3.97 (m, 1H, H_A_), 4.00 (dd, *J* = 11.6 Hz, 10.8 Hz, 1H, H_D1_), 4.09 (dd, *J* = 8.4 Hz, 6.8 Hz, 1H, H_A_), 4.17 (dd, *J* = 11.6 Hz, 2.6 Hz, 1H, H_D2_), 4.20–4.25 (m, 1H, H_B_), 4.54 (dd, *J* = 11.4 Hz, 2.6 Hz, H_E1_), 6.64 (dd, *J* = 9.2 Hz, 2.8 Hz, 1H, H_F_), 7.43–7.49 (m, 2H, H_K_), 7.50–7.54 (m, 1H, H_L_), 8.23–8.28 (m, 2H, H_J_) ppm. ^13^C-NMR (CDCl_3_, 100 MHz): δ 25.4, 26.4, 62.8, 65.0, 66.6, 74.1, 74.4, 81.9, 110.3, 115.2, 128.4, 130.1, 132.4, 137.5, 142.1, 144.9, 149.3, 157.2, 175.6 ppm. For the mixture of **8a** and **8b: **IR (neat): 3214, 2985, 1635, 1597, 1480, 1116, 1067, 1048 cm^−1^. HRMS (ESI): Calcd. for C_21_H_23_N_5_O_5 _[M+H]^+^ 426.1778, Found 426.1776. 

**Figure 4:**
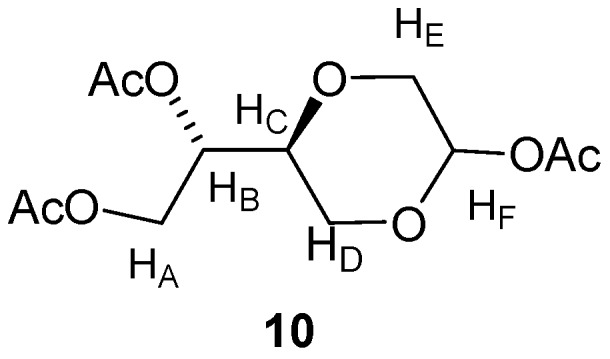
Structure of compound **10**.

*(2R,5S)-5-[(1S)-(1,2-Diacetyloxy)]-2-acetyloxy-1,4-dioxane* (**10a**) *and (2S,5S)-5-[(1S)-(1,2-diacetyl-oxy)]-2-acetyloxy-1,4-dioxane* (**10b**). Compound **3 **(2.2 g, 10.8 mmol) and *p*-TsOH (2.26 g, 11.9 mmol) was dissolved in acetic anhydride (50 mL). The mixture was stirred for 7 hours at room temperature and then poured into ice water. To the mixture was neutralized with sodium bicarbonate, and then extracted with ethyl acetate. The organic phase was dried over anhydrous MgSO_4_ filtration and evaporated under reduced pressure. The residue was purified by flash chromatography using a mixture of diethyl ether and *n*-hexane (2:1) as the eluent. The product appeared as yellow oil, (2.35 g, 75%). The diastereomers were not separated, however the NMR of the one diastereomer (Figure 4) was assigned the NMR signals: ^1^H-NMR (CDCl_3_, 400 MHz): δ 2.07, 2.14, 2.15 (s, 3 × 3H, CH_3_), 3.59–3.61(m, 1H, H_D_), 3.83 (dd, *J =* 12.8 Hz, 2H, 1H, H_E_), 3.89–3.98 (m, 3H, H_E_, H_D_ and H_C_), 4.16 (dd, *J =* 12 Hz, 6.8 Hz, 1H, H_A_), 4.36 (dd, *J =* 11.8 Hz, 4.2 Hz, 1H, H_A_), 5.15–5.18 (m, 1H, H_B_), 5.87 (d, 2Hz, 1H, H_F_) ppm. ^13^C-NMR (CDCl_3_, 100 MHz): δ 20.7, 20.8, 21.1, 61.2, 62.1, 68.1, 69.8, 73.6, 88.2, 169.8, 170.2, 170.5 ppm. The NMR data for the other diastereomer were the following:^ 1^H-NMR (CDCl_3_, 400 MHz): δ 2.10, 2.11, 2.16 (s, 3 × 3H, CH_3_), 3.78–3.82(m, 1H, H_C_), 3.83 (dd, 12.8Hz, 2Hz, 1H, H_E_), 3.93 (d, 12.8Hz, 1H, H_E_), 4.13–4.26 (m, 5H, H_B_, H_D _and H_A_), 5.87 (d, 2Hz, 1H, H_F_) ppm. ^13^C-NMR (CDCl_3_, 100 MHz): δ 20.7, 20.8, 21.4, 63.1, 63.4, 67.6, 68.8, 74.2, 89.0, 169.7, 170.5, 170.6 ppm. IR (neat): 2959, 1737, 1219, 1044, 1014 cm^−1^. MS (m/z): 289.3, 243.3, 231.3, 217.3. 

*N-6-Benzoyl-N-9-{(2R,5S)-5-[(1S)-(1,2-diacetyloxy)]-1,4-dioxan-2-yl}adenine* (**11a**) *and N-6-benzoyl-N-9-{(2S,5S)-5-[(1S)-(1,2-diacetyloxy)]-1,4-dioxan-2-yl}adenine* (**11b**). N-6-benzoyladenine (1.09 g, 4.6 mmol) and ammonium sulfate (62 mg, 0.47 mmol) in HMDS (50 mL) was refluxed overnight. The mixture containing the silylated N-6-benzoyladenine **7** was concentrated under reduced pressure, dissolved in dry dichloroethane (24 mL) and then added a solution of **10** (0.74 g, 2.6 mmol) in dry dichloromethane (6 mL). This mixture was added TMSOTf (0.94 mL, 5.2 mmol) at 0 °C and stirred overnight at room temperature. Chloroform (50 mL) was added to the mixture which was washed with saturated NaHCO_3_ solution (25 mL). The aqueous phase was extracted with chloroform (50 mL). The combined organic layers were dried over anhydrous Na_2_SO_4_ and filtered and concentrated under reduced pressure. The residue was purified by flash chromatography using a mixture of dichloromethane and methanol (9/1) as the eluent to afford the product (0.67 g, 56%) as a mixture of the *trans* and *cis* products, **11a** and **11b**. The product was used for the next reaction without further purification. The NMR was too complex to allow reasonable assignments of the signals. IR (neat): 2958, 1737, 1636, 1216.1045, 1023 cm^−1^. HRMS (ESI): Calcd. for C_22_H_23_N_5_O_7 _[M+H]^+^ 470.1677, Found 470.1663. 

**Figure 5:**
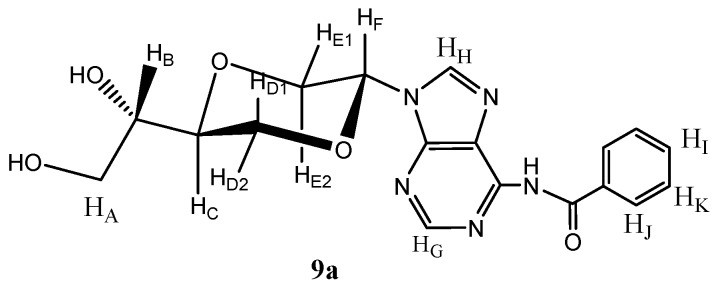
Structure of compound **9a**.

*N-6-Benzoyl-N-9-{(2R,5S)-5-[(1S)-(1,2-dihydroxyl)]-1,4-dioxan-2-yl}adenine* (**9a**) *and N-6-benzoyl-N-9-{(2S,5S)-5-[(1S)-(1,2-dihydroxyl)]-1,4-dioxan-2-yl}adenine* (**9b**). Compound **11 **(1.27 g, 30 mL) was dissolved in 7N ammonia in methanol (30 mL) and stirred for 7 hours at room temperature. The solution was concentrated and purified by flash chromatography using a mixture of dichloromethane and methanol (19/1) as the eluent. The pure *trans*- product **9a** was isolated (140 mg, 13.5%, Figure 5). Product **9a** exhibited the following spectroscopic properties: ^1^H-NMR (CD_3_OD, 400 MHz): δ 3.59 (dd, *J* = 11.2 Hz, 9.4 Hz, 1H, H_E2_), 3.60–3.70 (m, 3H, H_A_ and H_B_), 3.89 (dt, *J* = 10.6 Hz, 2.8 Hz, 1H, H_C_), 4.13 (dd, *J* = 11.8, 10.6 Hz, 1H, H_D1_), 4.24 (dd, *J* = 11.8, 2.8 Hz, 1H, H_D2_), 4.37 (dd, *J* = 11.2 Hz, 2.6 Hz, 1H, H_E1_), 6.55 (dd, *J* = 9.4, 2.6 Hz, 1H, H_F_), 7.41–7.45 (m, 1H, H_I_), 7.49–7.53 (m, 2H, H_K_), 8.21–8.24 (m, 1H, H_C_), 8.24 (s, 1H, H_G_), 8.67 (s, 1H, H_H_) ppm. ^13^C-NMR (CD_3_OD, 100 MHz): δ 63.8, 70.4, 70.8, 72.1, 76.3, 82.6, 115.5, 129.3, 131.0, 133.4, 138.2, 144.2, 145.2, 149.7, 156.9, 176.6ppm. IR (neat): 3286, 2876, 1635, 1424, 1285, 1115, 1063, 1021cm^-1^. HRMS (ESI): Calcd. for C_18_H_19_N_5_O_5 _[M+H]^+^ 470.1677, Found 470.1668. 

**Figure 6:**
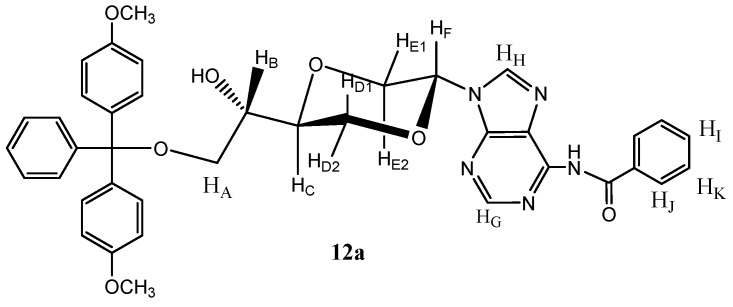
Structure of compound **12a**.

*N-6-Benzoyl-N-9-{(2S,5S)-5-[(1S)-hydroxyl-2-O-(4,4-dimethoxytrityl)-ethyl-1-yl]-1,4-dioxan-2-yl}-adenine* (**12**). Compound **9 **(mixture of **9a** and **9b**, 139 mg, 0.36 mmol) and dimethoxyltrityl chloride (277 mg, 0.79 mmol) were dissolved in dry pyridine (10 mL). The solution was stirred at room temperature for 3 hours. The solution was concentrated *in vacuo* and residue was purified by flash chromatography using first diethyl ether and then ethyl acetate as the eluent to provide the product (**12a** and **12b**) as a white solid (100 mg, 40%). Product **12a** (Figure 6) was obtained upon repeated chromatography and exhibited the following spectroscopic properties: ^1^H-NMR (CDCl_3_, 400 MHz): δ 2.82 (d, *J =* 4.8 Hz, 1H, OH), 3.25 (dd, *J =* 9.6 Hz, 5.4 Hz, 1H, H_A_), 3.34 (dd, *J =* 9.6 Hz, 5.6 Hz, H_A_), 3.43 (dd, *J =* 11.2 Hz, 9.2 Hz, H_E2_), 3.75–3.82 (m, 1H, H_B_), 3.77 (s, 2 × 3H, OCH_3_), 3.86–3.90 (m, 1H, H_C_), 4.00 (dd, *J =* 12 Hz, 10.2 Hz, H_D1_), 4.13 (dd, *J =* 12 Hz, 2.8 Hz, H_D2_), 4.43 (dd, *J =* 11.2 Hz, 2.8 Hz, H_E_), 6.60 (dd, *J =* 9.2 Hz, 2.8 Hz, H_F_), 6.82–6.86 (m, 5H, aromatic protons), 7.20–7.24 (m, 1H, aromatic proton), 7.26–7.37 (m, 5H, aromatic protons), 7.40–7.53 (m, 5H, aromatic protons), 8.13 (s, 1H, H_G_), 8.15–8.25 (m, 2H, aromatic protons), 8.64 (s, 1H, H_H_) ppm. ^13^C-NMR (CDCl_3_, 100 MHz): δ 55.2, 63.8, 69.3, 69.4, 70.0, 75.5, 81.0, 86.5, 113.2, 126.9, 129.8, 130.0, 132.3, 135.7, 135.8, 137.1, 142.1, 143.0, 144.6, 148.4, 157.1, 158.6, 175.4 ppm. HRMS (ESI): Calcd. for C_39_H_37_N_5_O_7 _[M+Na]^+^ 710.2591, Found 710.2588.

**Figure 7:**
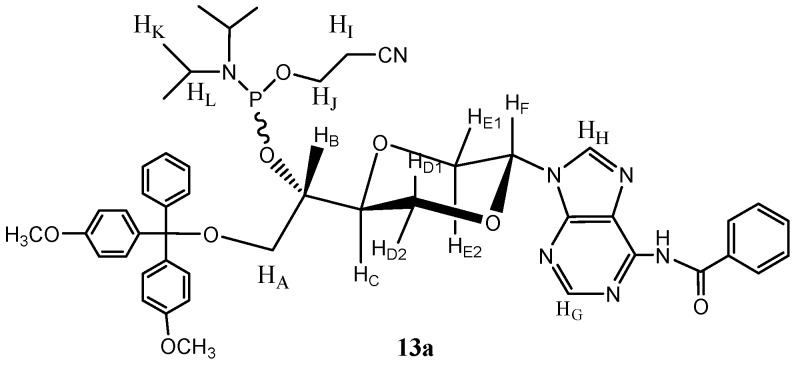
Structure of compound **13a**.

*N-6-Benzoyl-N-9-{(2S,5S)-5-{(1S)-O-[2-cyanoethoxy(diisopropylamino)phosphino]-2-O-(4,4′-di-methoxytrityl)-ethyl-1-yl}-1,4-dioxan-2-yl}adenine* (**13a**). Compound **12 **(mixture of **12a** and **12b**) (99 mg, 0.14 mmol) was dissolved in dry dicholormethane (6 mL) was then added *N,N*-diisopropyl-ethylamine (50 µL, 0.29 mmol). Then *N,N*-diisopropyl-2-cyanoethyl phosphoramidic chloride (105 µL, 0.47 mmol) was added. The resulting solution was stirred for 6 hours at room temperature. The solution was then concentrated and purified by flash chromatography using a mixture of ethyl acetate and n-hexane (4:1) as the eluent to give the *trans* isomer **13a** (65 mg, 51%) as a mixture of diastereomers due to the stereogenic phosphorus. Major diastereomer **13a** (Figure 7) had the following spectroscopic properties: ^1^H-NMR (CDCl_3_, 400 MHz): δ 1.14 (d, *J* = 7.2 Hz, 2 × 3H, H_K_), 1.19 (d, *J* = 7.2 Hz, 2 × 3H, H_K_), 2.64 (dt, *J* = 6.4, 2.4 Hz, 2H, H_I_), 3.27 (dd, *J* = 9.4, 5.2 Hz, 1H, H_A_), 3.36 (dd, *J* = 9.4, 6 Hz, 1H, H_A_), 3.45 (dd, *J* = 11.2, 9.4 Hz, 1H, H_E2_), 3.56–3.70 (m, 2 × 1H, H_L_), 3.771, 3.768 (s, 2 × 3H, OCH_3_), 3.72–3.82 (m, 1H, H_J_), 3.87–3.95 (m, 1H, H_J_), 4.00–4.09 (m, 3 × 1H, H_B_, H_D_, H_C_), 4.24 (d, *J* = 10 Hz, 1H, H_D_), 4.40 (dd, *J* = 11.2, 2.6 Hz, 1H, H_E1_), 6.61 (dd, *J* = 9.4, 2.6 Hz, 1H, H_F_), 6.81–6.85 (m, 4H, aromatic H), 7.19–7.22 (m, 1H, aromatic H), 7.27–7.30 (m, 2H, aromatic H), 7.34–7.36 (m, 4H, aromatic H), 7.41–7.48 (m, 4H, aromatic H), 7.52–7.56 (m, 4H, aromatic H), 8.14 (s, 1H, H_G_), 8.22–8.25 (m, 2H, aromatic H), 8.69 (s, 1H, H_H_), 12.59 (s, 1H, NH) ppm. ^13^C-NMR (CDCl_3_, 100 MHz): δ 20.4, 20.5, 24.56, 24.64, 24.67, 24.74, 43.2, 43.4, 55.2, 58.0, 58.2, 60.4 ,69.55, 69.62, 72.1, 75.3, 75.4, 81.3, 86.4, 113.1, 114.4, 117.7, 126.8, 127.8, 128.2, 128.3, 129.9, 130.05, 130.09, 132.3, 135.95, 136.01, 137.2, 141.8, 143.3, 144.8, 148.4, 157.2, 158.5, 175.5 ppm. ^31^P-NMR (CDCl_3_): 151.0, 152.0 (small) ppm. IR (neat): 3239, 3056, 2965, 2929, 2362, 2338, 1637, 1507, 1251, 1083, 788, 754, 719 cm^−1^. HRMS (ESI): Calcd. for C_48_H_54_N_7_O_8_P [M+Na]^+^ 910.3669, Found 910.3639.

**Figure 8:**
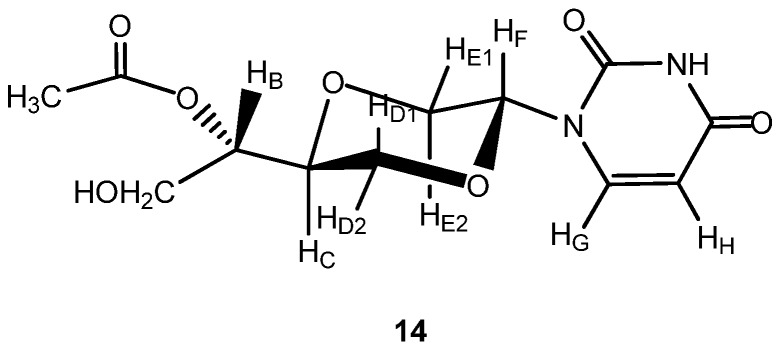
Structure of compound **14**.

*1-[(2R,5S)-5-[(1S)-Acetyloxy-2-hydroxylethyl-1-yl]-1,4-dioxan-2-yl]uracil* (**14a**). The synthesis of this compound has been reported earlier [7]. Compound **14a** (Figure 8) exhibited the following spectroscopic properties: ^1^H-NMR (CD_3_OD, 400 MHz): δ 2.07 (s, 3H, CH_3_), 3.58 (dd, *J* = 11.4 Hz, 9.8Hz, 1H, H_E2_), 3.70–3.74 (m, 1H, H_B_), 3.77–3.82 (m, 1H, H_C_), 3.92–3.98 (m, 2H, H_E_ and H_D1_), 4.06 (dd, *J* = 11.6, 2.8 Hz, 1H, H_D2_), 4.13 (dd, *J* = 11.2, 6.4 Hz, 1H, H_A_), 4.16 (dd, *J* = 11.2 Hz, 5.4Hz, 1H, H_A_), 5.69 (dd, *J* = 10 Hz, 2.8 Hz, 1H, H_F_), 5.70 (d, *J* = 8 Hz, 1H, H_H_), 7.71 (d, *J* = 8 Hz, 1H, H_G_) ppm. ^13^C-NMR (CD_3_OD, 100 MHz): δ 20.9, 66.4, 69.2, 69.4, 69.8, 76.0, 80.0, 103.1, 142.3, 151.9, 166.0, 172.8 ppm. IR (neat): 3477, 3190, 3110, 3074, 2996, 2879, 1697, 1268, 1105 cm^−1^. MS (EI): 230.3, 197(M^+^-(CH_3_COCHCH_2_OH)), 189(M^+^-uracil). HRMS (ESI): Calcd. for C_12_H_16_N_2_O_7_ [M+Na]^+^ 323.0856, Found 323.0862.

**Figure 9:**
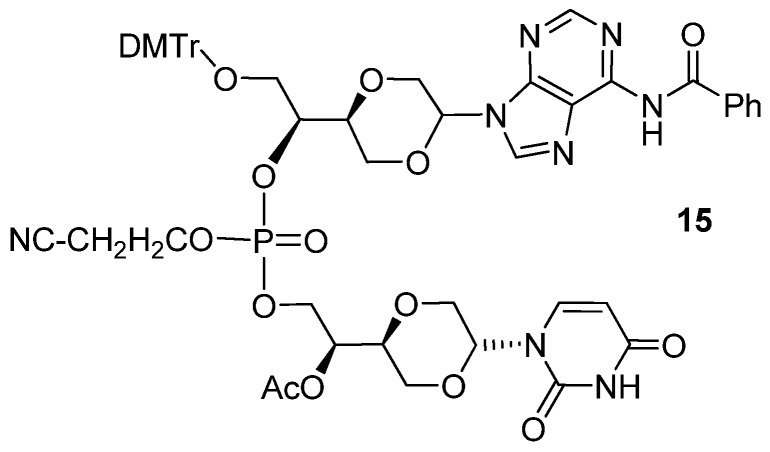
Structure of compound **15**.

*N6-Benzoyladenine uracil dinucleotide* (**15**). Compound **13a** (30 mg, 0.039 mmol) and **14a **(8 mg, 0.027 mmol) in a reaction flask with a magnetic stirring bar were dried under high vacuum for 4 hours. Then a mixture 1-*H* tetrazole (0.3 mL, 0.45 M, 0.135 mmol) in dry acetonitrile (6 mL) was added under a nitrogen atmosphere. The solution was stirred overnight at room temperature. Then was added a few drops of iodine in THF (1M solution), 2,6-lutidine and H_2_O (2:2:1) until an orange color persisted. The solution was then quenched with saturated sodium thiosulfate solution (4 mL). Then the two phases were treated with saturated NaHCO_3_ solution (4 mL). The separated aqueous phase was extracted with 4 × 8 mL of dichloromethane. The combined organic phase was dried over anhydrous Na_2_SO_4_ and filtered and the solvent evaporated. Flash chromatographic purification, using mixtures of dichloromethane and methanol in the ratio from 25:1 to 15:1 as gradient solvents, afforded a product (16 mg, 61.5%), which was assigned structure **15** (Figure 9) based on the following spectroscopic properties: ^1^H-NMR (CDCl_3_, 400 MHz): δ 2.02 (s, 3H, CH_3_COO), 2.09 (s, 3H, CH_3_COO), 2.51–2.62 (m, 2H, CH_2_CN), 2.71–2.74 (m, 2H, CH_2_CN), 3.15 (dd, 1H, *J* = 11.2 Hz, 9.6Hz), 3.21–3.28 (m), 3.38 (dd, *J* = 11.4 Hz, 9.8Hz), 3.45–3.58 (m), 3.62–3.96 (m), 4.01 (dd, 1H, *J* = 11.2 Hz, 2.8Hz), 4.03–4.13 (m), 4.14–4.35 (m), 4.37–4.47 (m), 4.63–4.74 (m), 5.58 (dd, 1H, *J* = 9.8 Hz, 3 Hz, anomeric proton), 5.66–5.71 (m, 3H, two protons in uracil and one anomeric proton), 6.62–6.68 (m), 6.84–6.89 (m, aromatic protons), 7.20 (d, *J* = 8 Hz, 1H, a proton in uracil), 7.22–7.28 (m, aromatic protons), 7.30–7.37 (m, aromatic protons and one proton in uracil), 7.42–7.49 (m, aromatic protons), 7.52–7.58 (m, aromatic protons), 8.143 (s, 1H, a proton in adenine), 8.148 (s, 1H, a proton in adenine), 8.23–8.25 (m, aromatic protons), 8.33 (br. 1H, NH), 8.63 (s, a proton in adenine), 8.64 (s, a proton in adenine) ppm. ^13^C-NMR (CDCl_3_, 100 MHz): δ 20.76, 20.82, 55.3, 62.0–62.5 (m), 67.6, 68.1, 68.4, 69.4, 73.2, 74.4, 74.8, 78.4, 78.5, 80.9, 86.8, 86.9, 102.7, 102.8, 128.06, 128.10, 128.3, 129.9, 130.0, 132.4, 134.98, 135.02, 135.2, 137.1, 139.2, 139.3, 141.9, 142.9, 148.4, 149.5, 157.2, 158.8, 162.1, 170.4, 175.6 ppm. ^31^P-NMR (CDCl_3_): −1.28, −1.49 ppm. HRMS (ESI): Calcd. for C_54_H_55_N_8_O_16_P [M+Na]^+^ 1125.3372, Found 1125.3354.

## 4. Conclusions

The synthesis of the adenosine analogs containing an optically active 1,4-dioxane sugar equivalent was achieved by coupling of *N*-silylated *N*-6-benzoyl protected adenine with the sugar acetate equivalents. Applying conventional phosphorimidite methodology the adenine analog was further coupled to the related uridine analog to give the corresponding protected dinucleotide, thus making it feasible that larger oligonucleotides can be prepared, including the application of conventional automated procedures.
